# Longitudinal integrated clerkships from start to finish: A medical curriculum innovation

**DOI:** 10.4102/phcfm.v16i1.4401

**Published:** 2024-04-27

**Authors:** Julia Blitz, Ian Couper, Maryke Geldenhuys, Marina Klocke, Maria van Zyl

**Affiliations:** 1 Centre for Health Professions Education, Faculty of Medicine and Health Sciences, Stellenbosch University, Bellville, South Africa; 2 Ukwanda Centre for Rural Health, Faculty of Medicine and Health Sciences, Stellenbosch University, Bellville, South Africa; 3 Division of Health Systems and Public Health, Faculty of Medicine and Health Sciences, Stellenbosch University, Bellville, South Africa; 4 Division of Family Medicine, Faculty of Medicine and Health Sciences, Stellenbosch University, Bellville, South Africa; 5 Centre for Disability and Rehabilitation Studies, Faculty of Medicine and Health Sciences, Stellenbosch University, Bellville, South Africa

**Keywords:** early clinical experience, longitudinal, continuity, holistic learning, professional identity formation

## Abstract

Stellenbosch University embarked on a renewal of its MBChB programme guided by an updated set of core values developed by the multidisciplinary curriculum task team. These values acknowledged the important role of (among others) context and generalism in the development of our graduates as doctors of the future for South Africa. This report describes the overall direction of the renewed curriculum focusing on two of the innovative educational methods for Family Medicine and Primary Health Care training that enabled us to respond to these considerations. These innovations provide students with both early longitudinal clinical experience (now approximately 72 h per year for each of the first 3 years) and a final longitudinal capstone experience (36 weeks) outside the central tertiary teaching hospital. While the final year experience will run for the first time in 2027 (the first year launched in 2022), the initial experience has got off to a good start with students expressing the value that it brings to their integrated, holistic learning and their identity formation aligned with the mission statement of this renewed curriculum. These two curricular innovations were designed on sound educational principles, utilising contextually appropriate research and by aligning with the goals of the healthcare system in which our students would be trained. The first has created opportunities for students to develop a professional identity that is informed by a substantial and longitudinal primary healthcare experience.

**Contribution:**

The intention is to consolidate this in their final district-based experience under the supervision of specialist family physicians and generalist doctors.

## Introduction

In 2018, Stellenbosch University started a process of university-wide curriculum renewal. As part of this project, the Faculty of Medicine and Health Sciences decided to attend to the MBChB programme. Consequently, we began responding to what we thought the South African healthcare system might need its new medical graduates to be like in 2028 and beyond. While focussing on the outcomes for this programme, we were also mindful of various trends in curriculum design, namely the student journey, including professional identity formation,^1^ the prevalence of mental distress among medical students^2^ and a shift to promoting wellness.^3^

At the initial workshops with a small group of health professions educationalists and committed teachers from a variety of foundational and clinical disciplines, we came up with a number of core values that we hoped would guide our thoughts in the design of such a curriculum. Among these were values that aligned well with the values of Family Medicine and that foregrounded Primary Heath Care (see Table 1).

**TABLE 1:** Core values of renewed curriculum (guiding principles).

Core value	Explanation
Contextual	Students will **understand the South African context, how our health system works, and their role within it**. They will recognise and be able to **engage with current societal needs** and participate in the positive transformation of healthcare in South Africa. A decolonised curriculum will value the contribution of diverse knowledge systems and literacies and will give all students equal opportunities to thrive and succeed.
Competence	The curriculum will facilitate the development of the necessary knowledge, skills and attitudes to become a competent doctor in our context. They will be able to integrate all six intrinsic graduate attributes roles into their professional practice. Students will have a future focus and will develop the necessary skills to use technology sustainably and effectively.
Core curriculum	Students will be trained as **generalists**; able to prevent, recognise and manage key health problems, and refer as appropriate. Selectives will provide students with opportunities to pursue individual interests.
Community	Students will **understand the social determinants of health and become active members of the communities they serve**; creating learning networks to impact health and disease. Decentralised training opportunities will allow for the development of ‘fit for purpose’ health professionals who can adequately and effectively respond to the health needs of the communities they serve.
Collaboration	Students will engage in **interprofessional, intersectoral and peer-based learning, collaboration and team work**.
Communication	Students will be able to communicate effectively in oral, written, digital and multi-modal forms in a variety of settings. They will develop multilingual, patient-centred, therapeutic and culturally appropriate communication skills to effectively elicit and share health information and engage in shared-decision making with patients, families and communities.
Compassion	Our curriculum will place the patient in the centre, and promote family centred, ethical practice. A **relationship-based model of care will promote partnership** and value-added dialogue towards building meaningful relationships.
Curiosity	Our students will be life-long learners who seek and practice evidence-based medicine. Through critical reflective practice, they will engage in self-evaluative learning to inform their continuous personal and professional development.
Critical and creative thinkers	The Stellenbosch graduate will be equipped to critically analyse and engage with problems, both simple and complex, with innovation. They will develop basic research skills and will interact and engage with the literature to inform their learning and practice.
Confidence	The Stellenbosch graduate will be and become a confident professional who displays a **high degree of self-efficacy**. Through **critical reflective practice,** they will develop a better understanding of the world and their place in it.
Care of self and others	Our students will experience a rich campus life and will **develop the necessary values and skills to not only take care of themselves** but also support the well-being of their peers, colleagues and teams. They will develop resilience, practice mindfulness and will have the emotional insight and imagination to understand the viewpoints of others.
Change agents	The Stellenbosch graduate will be accountable practitioners, who employ leadership and health advocacy competencies to effect positive change. They will have a **good understanding of health systems dynamics in order to drive health system changes**. They will be resource conscious and apply good stewardship of healthcare and environmental resources.
Citizenship	The Stellenbosch graduate will be engaged, committed and accountable agents of social good, who aspire to **contribute to social justice, democracy and equity**. As engaged citizens, they will understand that transformation of society involves transformation of the self.

Note: Those particularly relevant to primary health care and family medicine are in bold.

**MISSION STATEMENT** (2018): The experience of the MBChB future graduate will be filled with exciting opportunities to develop as a well-rounded, dynamic professional and team member with the ability to use the appropriate knowledge, skills and attributes in an innovative and relevant way for the South African healthcare reality and the world beyond.

**FIGURE 1:**
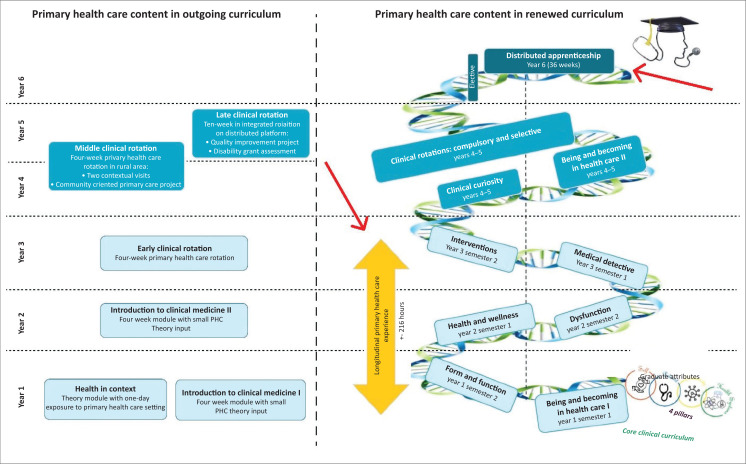
Outgoing curriculum PHC content (left) and renewed curriculum design (right).

After much work, and many conversations, an array of new approaches and ideas evolved, brought together in a double helix curriculum design map (see Figure 1), with one helix representing the core curriculum and the other the graduate attributes of the FMHS.^4^ The base pairs that hold the helices together represent continuous attention to the four pillars of self (the student), patient, community and the healthcare system. The foundational 3-year phase introduced six semester modules with a Longitudinal Primary Health Care Experience throughout. The intermediate phase will enable students to develop and apply their foundational knowledge while participating in clinical rotations in a tertiary setting, and in the final year, students will gain workplace-based experience in district and rural settings in the Distributed Clinical Apprenticeship. While the exit level outcomes of the renewed curriculum remain similar to the outgoing curriculum, we based our choices on work performed in the faculty on distributed and rural training,^5,6,7^ as well as modern evidence-informed educational processes.^8^ Educationally, we were guided by theories of integration of curricula, social learning, experiential and transformative learning, social justice and technology-enhanced learning and have responded to many of the recommendations in the 2010 Lancet Commission on transforming health professions education.^9^

We present here two of the major innovations in the curriculum that are pertinent to Family Medicine and Primary Health Care (PHC) training in Africa, marked with red arrows in Figure 1.

## Longitudinal primary health care experience

Small groups of students visit the same community-based primary health care facility on the same morning every week of the academic year from early in 1st year through to the end of 3rd year – a total of approximately 30 mornings or 72 h per year. They are part of a consistent group of MBChB I – III students for the whole year. Each week’s experience^10^ is preceded by training in team dynamics, language, clinical communication and appropriate procedural skills (in simulation). At the clinic and during visits in the surrounding community (schools, hospice, etc.), a PHC-experienced nurse facilitator supervises their practice and learning. This is guided by a logbook outlining expected tasks, practical procedures and individual and group assignments. These assignments – a Health Systems Project in Year 1, a Health Promotion Project in Year 2 and a Planetary Health Project in Year 3 – are aligned with the Western Cape Department of Health and Wellness vision of strengthening health promotion, disease prevention and primary health care using a Community Orientated Primary Care (COPC) approach.^11^ The facilitator will observe the student at work in the facility and give feedback with regard to his or her progress towards being able to perform logbook tasks independently using an adapted entrustability scale.^12^ Students from other undergraduate professional programmes may also be there, thus providing opportunities for interprofessional collaborative learning. At the end of their morning, they are asked to reflect on their learning using the 3Ds (delights, difficulties and discoveries – considering what they had found delightful, what had been difficult and what they had discovered regarding themself, the patient, the community and/or the health system). Every 6 weeks, they will use these reflections and their facilitator feedback forms during their progress check-in with their student mentor.

In terms of our curriculum design, this early clinical experience attends to a number of our goals. In terms of the four pillars of the curriculum, it enables the student to explore the primary health care system while gaining an understanding of the patient’s experience in the context of the surrounding community. The reflections assist the students to monitor their professional identity formation as they mature as medical students. The experience makes the core curriculum of common presenting symptoms visible (the curriculum walks through the door), providing students with the opportunity to apply their foundational science learning and develop clinical reasoning skills while also embedding multi-disciplinary and inter-professional collaborative practices that had always been part of the PHC components of the curriculum.

Educationally, this experience is based on the principles of longitudinality^13^ and continuity,^14^ offering students the opportunity to be embedded in clinical teams and learning groups with the added value of near-peer orientation and teaching and support from more senior students in the group.^15^ This capitalises on social cognitive learning theory.^16^ The context of primary care (with fewer clinically complex patients than in a tertiary hospital) allows early clinical experience to support students through staged and appropriate experience-based learning.^17^ Such experience helps medical students socialise with their chosen profession and assists in making their learning more real and relevant.^18^ The unavoidable proximity to communities enables the students to reflect on the realities of social injustice and health inequities,^19^ linked to social determinants of health and sustainable development goals.

## Distributed clinical apprenticeship

The plan is for all final year students to spend the full duration of their final year outside of our traditional tertiary or quaternary academic hospital complex, working and learning in primary and secondary care under the supervision of specialist family physicians, general specialists and generalist doctors across the distributed teaching platform.^18^ After an elective period, students will spend the remainder of their 6th year (36 weeks) in one health ecosystem (this varies but would usually be a health subdistrict or an entire health district), which will offer exposure to a range of opportunities that will enable the transition to internship and ensure they achieve the kind of graduate attributes envisioned by the overall curriculum plan. Again longitudinality and continuity are important design features as the group of students embed within clinical teams at the sites and are given supervised responsibility for patient care^20^ – truly working as apprentices alongside qualified healthcare practitioners. A cohort of students will have the opportunity of selecting to do their DCA as a Longitudinal Integrated Clerkship,^21^ an approach increasingly adopted internationally for its educational benefits^22^ and workforce outcomes^23^ and which has been an option within our Rural Clinical School (RCS) since 2011 and from which we have learnt important lessons.^24^

Also building on the RCS experience, a student’s learning portfolio, including their logbook of procedures, will drive self-regulated learning towards achieving competency-based outcomes. Students (and practitioners) will be able to access technologically enhanced support from specialists at the central hospital complex. Students will be deemed to have completed sufficient disciplinary knowledge and skills assessments at the end of 5th year, so that the focus of assessment can now move to workplace-based formats, including completion of projects that make a difference to the community and/or hospital, such as a quality improvement project. The four pillars of the curriculum helix will again form a strong basis for students’ activities.

## Conclusion

So what was the outcome of these innovations? It is a little early to tell, as the renewed curriculum only enters its third year in 2024 and students will not reach the Distributed Apprenticeship until 2027. Faculty budget cuts and the high cost of salaries and transport have resulted in a 50% reduction of the originally envisaged weekly early clinical learning experience to every second week. The heterogeneity of facilities and learning opportunities on the platform has resulted in assigning students to more than one facility within the same community (geographical training zone) over the course of the three years. As with all curriculum innovation, the tension is how to make best use of limited resources. This may be resolved by defining the goals for one’s students. While students are supervised by the same PHC facilitator throughout the first 2 years, the need to develop their clinical reasoning, diagnostic and management skills will see their clinical learning experience in Year 3 being facilitated by an on-site medical practitioner.

At times, students have been frustrated by waiting for their shuttle transport. But their evaluations have been overwhelmingly positive, with some common themes being a sense of appreciation for the space to engage with patients and communities so early in their journey of becoming a healthcare professional, the supportive relationship with longitudinal primary health care experience (LPHCE) facilitators and the opportunity to engage in holistic and relevant learning.

As with all curriculum innovation, the tension is how to make best use of limited resources. This may be resolved by defining the goals for one’s students. We believe that if we want graduates who have experience of learning in primary (and secondary) healthcare in South Africa, and who therefore develop the confidence and skills to practice at those levels, it requires a deliberate redistribution of resources to enable such innovations.

We are confident that our radical shift in approach and a much greater focus on primary health care, will produce a different graduate who is ready and equipped to support the goal of delivering universal access to healthcare in our country.
